# Draft Genome Sequence of a New Delhi Metallo-β-Lactamase-5 (NDM-5)-Producing Multidrug-Resistant Escherichia coli Isolate

**DOI:** 10.1128/genomeA.00017-15

**Published:** 2015-03-05

**Authors:** Tom J. B. de Man, K. Allison Perry, Johannetsy J. Avillan, J. Kamile Rasheed, Brandi M. Limbago

**Affiliations:** Division of Healthcare Quality Promotion, Centers for Disease Control and Prevention, Atlanta, Georgia, USA

## Abstract

A multidrug-resistant Escherichia coli isolate from an abdominal lesion displayed resistance to all β-lactams tested, including carbapenems, in addition to macrolides, fluoroquinolones, and tetracycline. Sequence analyses demonstrated the presence of *bla*_NDM-5_ in addition to at least 13 genes and 6 efflux pumps associated with antibiotic resistance.

## GENOME ANNOUNCEMENT

Multidrug-resistant Gram-negative bacteria are an increasing public health threat, particularly the emergence of carbapenem-resistant *Enterobacteriaceae* (CRE). Limited therapeutic options exist for infections caused by CRE, which can be associated with high mortality (1, 2). Although carbapenem resistance may result from a variety of mechanisms, the most concerning is the production of carbapenemases, including Klebsiella pneumoniae carbapenemase (KPC), the most common carbepenemase among *Enterobacteriaceae* in the United States (3). The recent emergence of the New Delhi metallo-β-lactamase (NDM) is of great concern globally. Whole-genome sequencing (WGS) is becoming an important tool for providing the capacity to detect emerging variants of known resistance genes as well as novel antibiotic resistance mechanisms through surveillance (4).

Numerous multidrug-resistant Escherichia coli isolates were obtained during the course of an investigation into health care-associated transmission in a tertiary care facility (5). All 39 outbreak-related isolates were very closely related (>92% similar) by pulsed-field gel electrophoresis (PFGE) and by WGS analysis (range, 1 to 28 single nucleotide polymorphisms in the core genome). One isolate, E. coli 1400026, obtained from an abdominal lesion, was selected for the annotation described here. DNA was extracted using the Maxwell 16 (Promega, Madison, WI) instrument and the Maxwell 16 Cell LEV DNA purification kit from overnight growth cultured on blood agar.

WGS was performed using an Illumina MiSeq and generated paired-end reads of 250 bp with 120-fold coverage on average. Reads were assembled *de novo* into contigs and subsequently joined into scaffolds by means of CLC Genomics Workbench 7.0.4. The draft genome yields 125 contigs with an *N*_50_ of 137,218 bp and a total assembly length of 4,980,047 bp.

*In silico* multilocus sequence typing (MLST) analysis, using the scheme by Wirth et al. (6), showed that this organism falls within sequence type 167. This internationally disseminated clone is associated with numerous resistance mechanisms including *bla*_NDM_, *bla*_CTX-M_, and *bla*_CMY_ (7–9).

Gene prediction was performed using Glimmer (10) and tRNAScan-SE (11). Additionally, RNAmmer (12) identified rRNA genes and Resfinder 2.1 (13) and a custom gene detection tool detected acquired antibiotic resistance mechanisms. Using an identified 16S gene as a BLAST query, the Enterotoxigenic (ETEC) H10407 Escherichia coli genome was identified as the closest relative and utilized as a comparator for annotating putative genes. We identified 4,817 protein genes, 76 tRNAs, and 3 rRNA genes (5S, 23S, and 16S). Our analyses revealed genes conferring resistance to several classes of antimicrobials including β-lactams (*bla*_NDM-5_ carbapenemase, *bla*_CTX-M15_ and *bla*_TEM-1_ extended spectrum β-lactamases, plasmid-mediated *bla*_CMY-42_, an ampH and *bla*_OXA-1_ β-lactamase), aminoglycosides (*aadA5*), aminoglycosides/fluoroquinolones acetyltransferase (aac(6′)-Ib-cr), tetracycline (*tetA*), macrolides (*mphA*), trimethoprim (*dfrA17*), chloramphenicol (*catB3*), a penicillin binding protein, and sulfonamides (*sul1*).

Six multidrug efflux systems were predicted, including three putative pumps, an EmrKY-TolC pump, and two macrolide pumps (macB and a macrolide specific pump).

The availability of this genomic sequence enables further comparative genomic analyses within E. coli strains and also provides information on the genetic background of antibiotic resistance.

### Nucleotide sequence accession numbers.

This whole-genome shotgun project has been deposited at DDBJ/EMBL/GenBank under the accession no. JTKA00000000. The version described in this paper is version JTKA01000000.
